# Laser as a toolbox for wood processing and functionalization

**DOI:** 10.1557/s43577-026-01063-5

**Published:** 2026-04-27

**Authors:** Yong Ding, Christopher H. Dreimol, Mélanie Rouèche, Armin Stumpp, Ronald Holtz, Chris Zhou, Orlando J. Rojas, Ingo Burgert

**Affiliations:** 1 Wood Materials Science, Institute for Building Materials, ETH Zürich, 8093 Zurich, Switzerland; 2 WoodTec Group, Cellulose & Wood Materials, Empa, 8600 Dübendorf, Switzerland; 3 Institute of Product and Production Engineering (IPPE), University of Applied Sciences and Arts Northwestern Switzerland (FHNW), Windisch, Switzerland; 4 Bioproducts Institute, Department of Chemical and Biological Engineering, The University of British Columbia, Vancouver, Canada; 5 Department of Wood Science, The University of British Columbia, Vancouver, BC V6T 1Z4 Canada

**Keywords:** Functional, Heterostructure, Laser, Multiscale, Biomaterial

## Abstract

Wood is a unique natural resource widely utilized for various applications, and its processing has a significant role in enabling its sustainable development. Laser technology has emerged as a powerful and precise tool for the functionalization and processing of wood materials, offering flexibility and sustainability benefits. This article explores the principles of laser–wood interactions, covering photothermal, photochemical, and photomechanical processes. We identified key parameters that influence laser processing efficiency, such as laser pulse, laser wavelength, and wood material composition. It highlights novel applications of laser processing, from enhancing permeability through laser cutting or drilling, to enabling it with new properties or functions via surface laser treatment technologies such as laser-induced graphene. Furthermore, the review discusses future perspectives of laser-assisted wood engineering, highlighting its critical role in driving sustainable practices and innovations in wood materials technology.

**Graphical abstract:**

**Figure:**
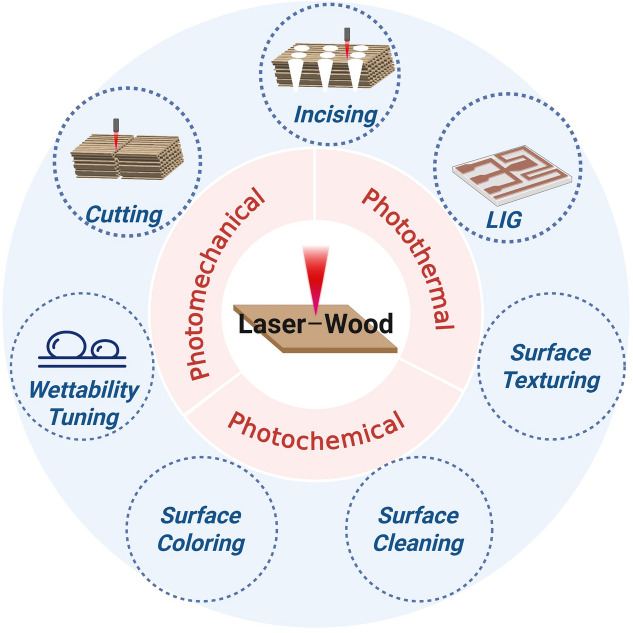
Overview of laser-wood interaction categories and applications. LIG, laser-induced graphene.

## Introduction

The pressing global challenges of climate change and fossil resource depletion drive an urgent need for more sustainable development. Biomass, particularly lignocellulosic materials, comprising cellulose, lignin, hemicelluloses, and resulting biocomposites such as wood, bamboo, as well as other agricultural residues, have become an attractive target for addressing these challenges. Among these, wood stands out uniquely, offering significant potential for influencing the engineering materials sector and advancing toward a bioeconomy.^1,2^ The intrinsic CO_2_-sequestration capability of trees inproducing wood, coupled with wood's outstanding mechanical properties, makes it a sustainable and technologically performant material for the building sector. Thus, responsible utilization of wood can help mitigate the ongoing climate change crisis.

Conventional modification methods, such as chemical or thermal treatments, were employed primarily to mitigate inherent limitations through surface or bulk (impregnation) treatments. Recently, intensive research has focused on functionalizing wood to enable innovative applications by modifying its structure and chemistry down to the cell wall level.^3,4^ Nevertheless, the effectiveness of these modifications greatly depends on factors such as impregnation efficiency. Conventional methods, such as vacuum and pressure treatments, are known to have limits in impregnating some of the wood species highly in demand, such as spruce.^5^

Emerging solutions to these challenges lie in advanced machining techniques that are minimally invasive and preserve the natural integrity of wood, such as electron-beam machining,^6^ abrasive water jet machining,^7^ and laser-beam machining. This latter technique, laser-beam machining has gained increasing popularity in wood science because of the high processing resolution and scalability.^8^ From intricate engravings to precise cuts and even surface treatments down to the microscopic level, laser processing holds significant potential for enhancing wood engineering.^9–13^ This article aims to provide an overview of underlying laser–wood interactions and advanced laser processing applications, offering insights into future research challenges in the emerging field of laser processing of wood and wood-based materials.

## Advantages and challenges of laser processing for wood materials

Laser-beam machining features high speed of operation, flexibility, and allows localized processing, reaching comparatively low surface roughness values. Here, we highlight the key advantages and challenges of processing wood materials with a laser.

Laser-based methods provide precise, noncontact processing, enabling targeted modification of lignocellulosic materials without physical tool wear or direct mechanical stress.^14,15^ This contact-free approach significantly reduces mechanical damage, enabling fine control over surface and internal microstructures, and preserving the intrinsic mechanical and porous structures of the substrate. The spatial resolution of lasers can be adjusted down to micrometers or even submicrometers scales, depending on wavelength and pulse duration. This capability enables intricate cutting, drilling, engraving, surface texturing, and patterning not achievable by conventional tools.

Moreover, laser processing is recognized for its economic and environmental benefits, notably low material waste, reduced chemical consumption, and scalability.^16,17^ Due to the narrow kerf widths achievable with laser cutting, waste generated from the process is minimal compared to most mechanical methods. Additionally, lasers require no pre- or posttreatment, simplifying downstream processing. Laser processing integrates well with digital and automated manufacturing. As Industry 4.0 practices expand, lasers facilitate digital customization and on-demand production, enhancing manufacturing responsiveness to market changes and promoting innovation in sustainable product development.^18^

However, laser–wood interaction can also produce undesirable surface effects. Heat-affected zone (HAZ) control is one key challenge in laser processing of wood, leading to undesired or degraded performance such as carbonization, discoloration, microcracks, and even burning. Generally, HAZ formation is governed by (1) light absorption; (2) thermal diffusion length ${L}_{\mathrm{th}}\sim \sqrt{\upalpha {\hspace{0.17em}}\uptau }$, with $\upalpha$  the thermal diffusivity and $\uptau$ the effective pulse; and (3) the reactive environment.^19^ The HAZ is also directly linked to fire and afterglow risk. Wood, as a lignocellulose material, shows strong absorption of a CO_2_ laser. Under oxygen environment, strong carbonization is promoted. A porous, carbonized char layer can remain hot and continue oxidizing after the beam passes, particularly in thick sections, resin-rich regions.

Safety considerations present another key challenge as smoke, particulate matter, and volatile organic compounds (VOCs) can be generated due to photothermal decomposition of cellulose, hemicelluloses, lignin, and extractives. These emissions are strongly process-dependent and can be influenced by shifting the local energy balance between evaporation, pyrolysis, and flaming. Accordingly, adequate local exhaust ventilation and filtration are essential, particularly for CO_2_-laser cutting/engraving where carbonization and soot formation are common.

Anisotropy and heterogeneity of wood present another key challenge from the material aspect. Wood is a hierarchical and heterogeneous material. The porosity and density can vary from wood species and between earlywood/latewood. These variations change local absorptivity and heat transport, producing uneven kerf geometry, local charring, and variability. Therefore, the advantages of laser processing are achieved when the HAZ or VOC emission is kept minimal and when material heterogeneity is well managed. This motivates the section on “Laser–wood materials interactions regimes,” where we outline the photothermal, photochemical, and photomechanical pathways that govern wood’s response to laser.

## Laser–wood materials interactions regimes

Building on the advantages and challenges previously discussed, we summarize the governing interaction regimes that determine processing outcomes. Laser processing utilizes the energy of a laser beam to interact with a material. When a laser-beam energy is received by a lignocellulosic substrate, the energy can drive a variety of processes in the material, from heating and evaporation to chemical bond breaking or even plasma formation, causing changes in structure or composition.^9,20–24^ Generally, laser–material interactions can be assigned to three reaction categories:

- *Photothermal*—conversion of photon energy to heat, leading to thermal reactions (e.g., pyrolysis, combustion).
- *Photochemical*—direct photonic excitation of chemical bonds, leading to bond scission or molecular modifications.
- *Photomechanical*—generation of mechanical forces via rapid heating, vaporization, or plasma expansion, which can cause fracture.

**Figure 1:**
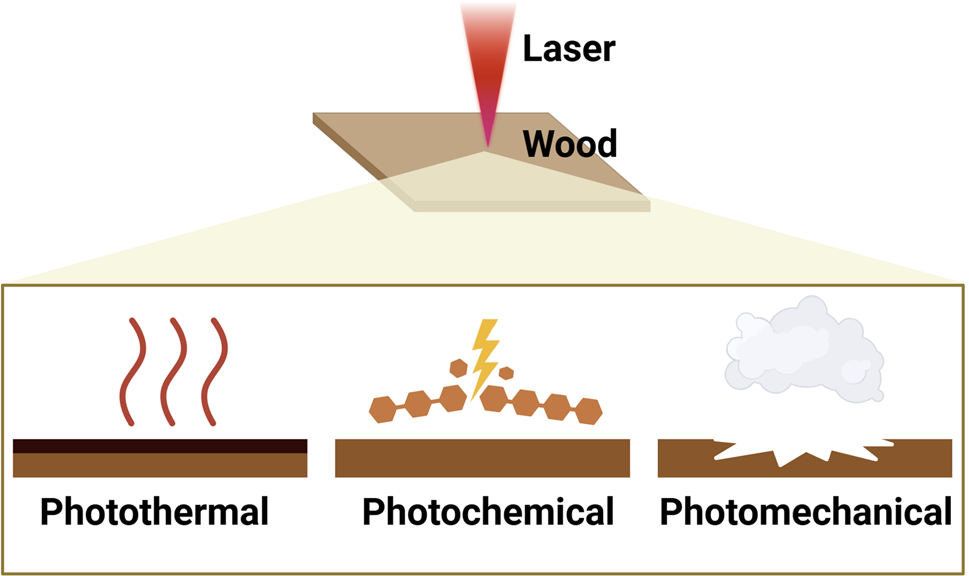
Three interaction categories between laser and wood: photothermal, photochemical, and photomechanical.

In practice, these categories often overlap, but it is useful to discuss each in turn with emphasis on the context of lignocellulosic substances (**Figure** 1).

### Laser–material interaction categories

#### Photothermal effects

For most lasers in the infrared and millimeter-wave range, the interaction with lignocellulose is primarily photothermal. Wood and other biomass strongly absorb IR radiation. For example, a CO_2_ laser at 10.6 µm deposits energy almost entirely as heat within the surface layers.^25^ This intense localized heating causes pyrolysis/combustion of the organic components in the presence of oxygen. The chemistry of laser-induced pyrolysis/combustion in wood mirrors that of conventional thermal treatment of biomass.

Below 200°C, bound and free water evaporate, and slow scissions in lignin, cellulose, and hemicellulose may begin, releasing predominantly noncombustible gases (water vapor). Around 300°C, initial char formation is typically observed. Between ~280 and 500°C, lignin, the aromatic binder in wood, is relatively thermally stable but eventually breaks apart and condenses into char, forming cross-linked carbonaceous structures while losing functional groups (e.g., methoxy (–OCH_3_) groups are cleaved in a process known as demethoxylation).^26^ In parallel, the polysaccharide components (cellulose and hemicelluloses) undergo depolymerization.^27–29^ Hemicelluloses begin to decompose at slightly lower temperatures than crystalline cellulose, both generally >300°C. Their breakdown yields volatile products (e.g., furfural and other dehydrated sugar derivatives), which can further condense into tarry compounds that impart a dark coloration to the heated wood.^26,30^ At sufficiently high energy densities, the entire surface layer carbonizes into a black char coat. The depth of this HAZ can be on the order of millimeters for a high-power CO_2_ laser, as heat conducts into the bulk.^31,32^ Managing the photothermal effect via laser parameters or material pretreatments is therefore crucial in laser cutting or engraving of wood to minimize unwanted HAZ.

#### Photochemical effect

At shorter wavelengths in the visible and ultraviolet (UV), laser–material interactions can enter a photochemical regime. Photons at high-energy states (e.g., UV photons > 4 eV) can directly excite and break specific chemical bonds in the material. Cellulose biopolymers absorb strongly in the UV spectrum. For example, Kolar et al. showed that a pulsed XeCl excimer laser (308-nm wavelength) drastically reduces the degree of polymerization of cellulose (cleaving its long chains), whereas a near-IR Nd:YAG laser at 1064 nm under similar conditions causes almost no depolymerization.^33^ These distinct effects arise from different laser–material interactions. While the UV photons directly break the C–O and C–C bonds of the cellulose (a photochemical effect), 1064-nm photons mainly generate heat (a photothermal effect) that can even promote cross-linking reactions instead of chain scission.^34,35^

By tuning wavelength and pulse parameters, one can favor direct bond-breaking over bulk heating, thereby achieving high-resolution ablation of cellulose with less HAZ.^36^ For instance, UV laser pulses at 355 nm have been used to pattern wood and paper surfaces by carbonizing only a very thin surface layer into conductive carbon.^37^ Because the penetration depth of UV in these materials is low, the laser energy is confined to the immediate surface, and the thermal diffusion is minimal. The tradeoff is that UV lasers typically have lower power and ablation rates, so they are used for fine, shallow features rather than deep cuts in thick wood.

#### Photomechanical effect

When laser intensities are very high or pulses are extremely short, they can create plasma at the surface as the material vaporizes and ionizes.^38,39^ The temperatures in a laser-induced plasma can exceed ~2000–2500 K. These extreme conditions lead to expulsion of molten or vaporized matter, generating pressure waves that can shock the surrounding material.^40^ Previous study showed that high-fluence nanosecond and femtosecond lasers can vaporize moisture in wood and create a shock that helps blow out material from a kerf, achieving a more mechanical cutting action as opposed to slow burning.^41^ Short pulses or high peak power lasers that drive ablation via plasma formation and shock waves can promote the photomechanical effects in the processing of lignocellulosic materials.

### Laser processing efficiency

In many practical scenarios, interaction regimes occur simultaneously. The efficiency of the process can be influenced by both the laser parameters (wavelength, pulse duration, intensity) and the material properties (composition, density, moisture content, etc.).

#### Influence of laser parameters

**Figure 2:**
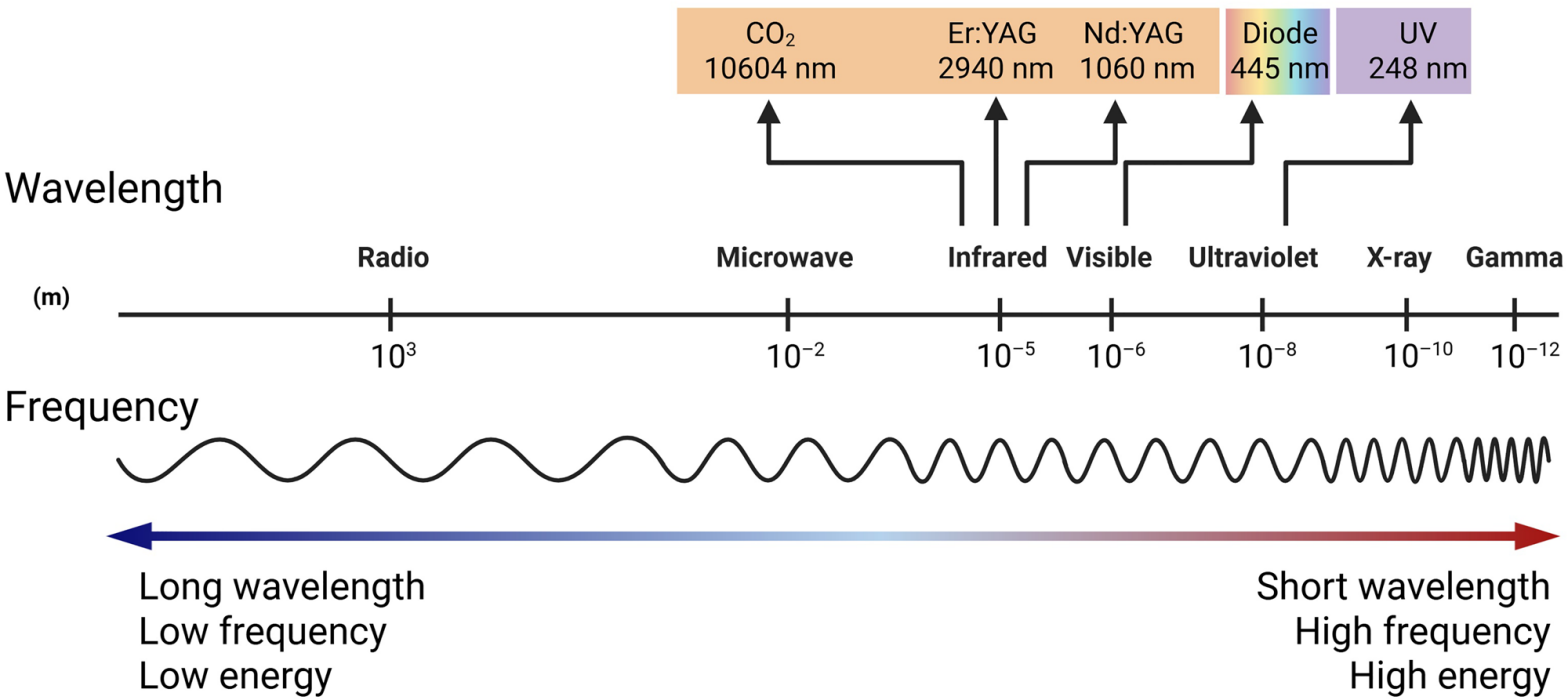
Wavelength and frequency of common lasers for wood processing.

Laser wavelength and pulse duration are critical factors that determine which of the above regimes dominate (**Figure** 2).^36^ Wavelength governs the absorption depth and the available photon energy. Long wavelengths (far IR, such as CO_2_ 10.6 µm) tend to penetrate a bit deeper but are efficiently absorbed by organic bonds, leading primarily to photothermal heating.^35,42,43^ Visible and near-IR wavelengths (e.g., 1 µm from fiber lasers) are less strongly absorbed by dry wood unless assisted by colored additives or by burning/sooting that increases absorption.^44,45^ In short, infrared lasers deliver heat deep into lignocellulose with a high risk of charring, whereas UV lasers deliver energy shallowly and can cleave molecules directly enabling “cold” ablation.

Pulse duration is another key parameter. Continuous-wave (CW) or long-pulse lasers (millisecond to microsecond regime) allow heat to conduct during illumination, yielding a wide HAZ and significant thermal degradation around the cut.^35^ By contrast, nanosecond or femtosecond pulses deposit energy faster, leading to localized energy deposition.^46,47^ For example, Costa et al. reported the use of ultrafast IR and UV pulses to bacterial nanocellulose and found no signs of thermal degradation in the material, confirming that material removal was via direct ablation rather than slow burning.^48^

#### Influence of material properties

The composition and microstructure of the wood substrate heavily determine the outcome of laser processing.^49^ Important factors are the lignin content and the density of the material. Wood species that are very dense and rich in lignin generally tend to char and carbonize more readily under laser heat.^50^ Another critical material parameter is moisture content. Water present in wood has a dual influence on laser interactions.^49,51–53^ On the one hand, water strongly absorbs certain IR wavelengths. As a result, wet or “green” wood often requires higher laser energy input to cut through, because a portion of the energy is diverted to the latent heat of vaporization of water.^54^ On the other hand, the presence of moisture can mitigate charring and improve the cut quality. The evaporating water acts as a heatsink and can carry away heat via the steam, preventing the wood temperature from soaring much above 100°C in the early stages of irradiation. This leads to less localized overheating.^52,53^ Thus, there is a tradeoff: moisture makes laser cutting less energy-efficient, but can act as a natural anti-char agent.

Awareness of these effects is important for optimizing laser processing of wood. **Table** I summarizes representative laser types and their interaction characteristics on lignocellulosic substrates. By understanding the principles of interactions, laser processes can be tailored from gentle surface modifications to ablation or carbonization. The next sections will explore how these interaction regimes are leveraged in various applications.

**Table I:** Representative laser processing regimes for lignocellulosic materials: typical conditions and observed effects.

Laser Type and Parameters	Potential Material	Observed Effects	References
CO_2_ laser (10.6 µm, continuous or ms-pulsed, tens of W)	Solid wood, bamboo	Photothermal heating; significant pyrolysis and char formation, deep heat-affected zones due to strong infrared absorption	Barcikowski & Ostendorf, 2006^55^
Er:YAG laser (2.94 µm, ms pulses)	Wood tissues	Strong absorption by moisture, combined photothermal and photomechanical mechanisms, precision drilling/incisions	Grad & Možina, 1998^56^
Nd:YAG/Fiber IR laser (1.06 µm, ns pulses, 10–100 W)	Wood, paper, natural fiber fabric	Primarily photothermal, reduced heat-affected zone; fine cutting and moderate charring possible due to lower absorption	Hernández-Castañeda et al., 2011^51^
Femtosecond laser (800 nm, ~100 fs pulses)	Wood, cellulose-based materials	Nonthermal, photomechanical ablation via plasma generation, negligible thermal damage, high precision at microscale	Naderi et al., 1999^57^ Costa et al., 2024^48^
Diode laser (445-nm blue, CW, <10 W)	Light wood, paper	Moderate absorption, primarily photothermal; smaller focal spot size (~0.1 mm), achieves fine detail engraving, moderate charring similar to other CW lasers	Leone et al., 2009^58^ Koukouviti et al., 2023^59^
UV laser (248-nm excimer, ns pulses)	Paper (cellulose), thin wood veneer, lignin film	Dominantly photochemical ablation, direct molecular bond cleavage, minimal thermal degradation, limited charring	Panzner et al., 1998^60^ Kolar et al., 2000^33^ Mahmud et al., 2018^61^ Jeong et al., 2020^37^

## Applications of laser processing in wood materials

**Figure 3:**
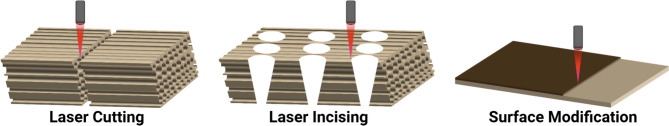
Illustration showing main laser processing techniques to structurally and chemically modify wood.

Laser can deliver contactless and localized treatments, which makes it particularly attractive for advanced manufacturing and functionalization of lignocellulosic materials. Laser processing has opened up diverse opportunities to modify wood and wood-derived materials. Next, we highlight specific laser techniques for structural and chemical wood modifications (**Figure** 3).

### Laser cutting and incising

#### Laser cutting

**Figure 4:**
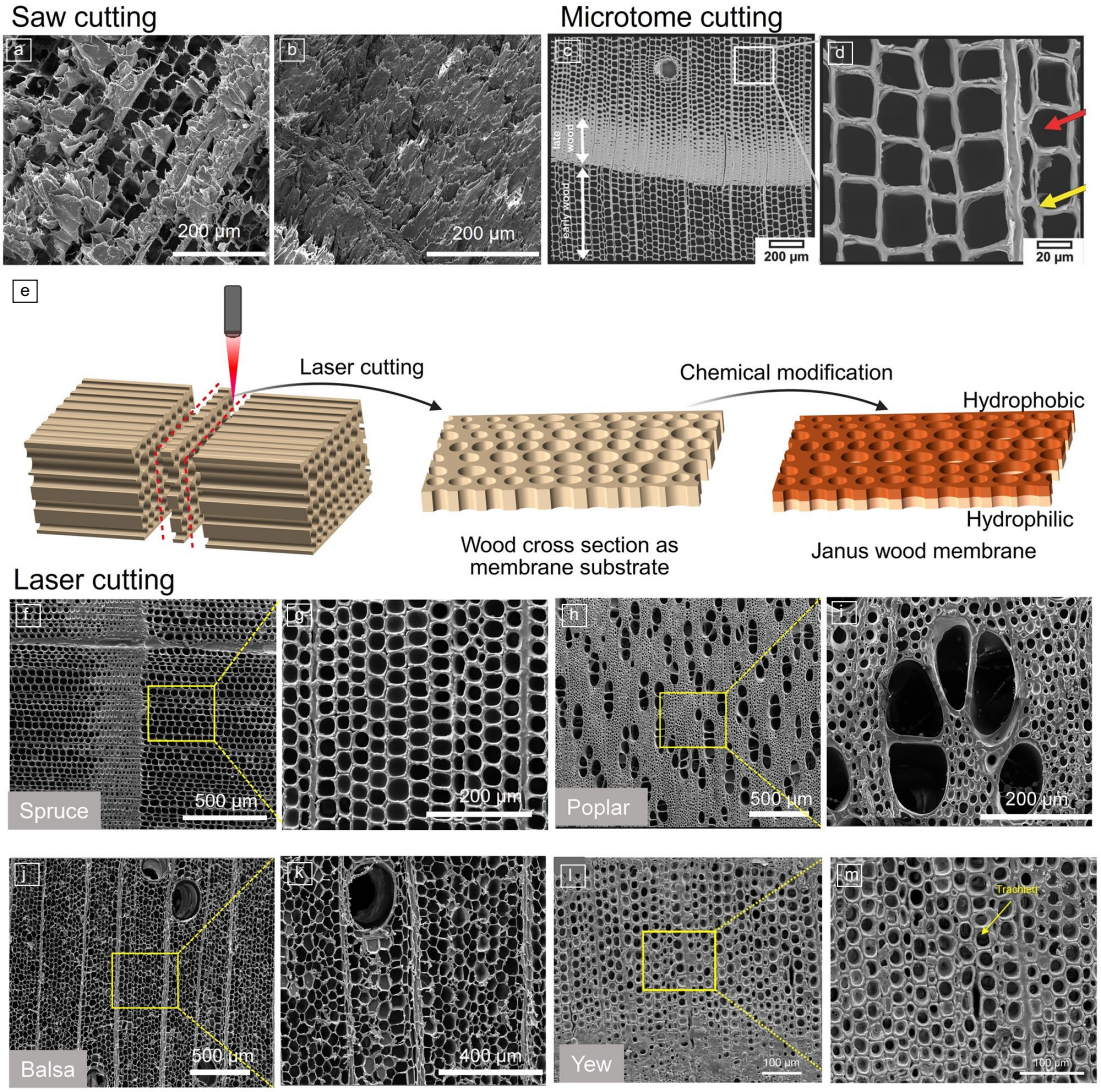
Comparison of wood cross sections produced by different cutting methods. Morphology of (a, b) saw-cut cross section of wood (Norway spruce) (published under license CC BY 3.0)^83^ and (c, d) microtone cut (© 2016, Wiley).^86^ (e) Illustration of the fabrication process: native wood membrane prepared by laser cutting then chemically modified. (f–m) Scanning electron microscopy images of the laser-cut cross sections of different wood species, (f, g) Norway spruce (*Picea abies*) (published under license CC BY 3.0),^83^ (h, i) poplar (*Populus *spp.) (published under license CC BY 4.0),^84^ (j, k) balsa (*Ochroma pyramidale*), (l, m) European yew (*Taxus baccata*) (© 2021, Elsevier).^85^

Laser cutting is particularly efficient in producing smooth cut surfaces of wood and similar materials with hierarchical porous structures, facilitating various applications requiring controlled fluid transport.^3,62–73^ Some common mechanical methods (e.g., sawing, planing, grinding) typically result in wood surfaces characterized by clogging and dust contamination due to partial destruction of the wood cell walls^74–77^ (**Figure** 4a–b). While microtome cutting can achieve much smoother surfaces, it is hardly applicable for thin wood cross sections (<3 mm)^78,79^ (Figure 4c–d).

Laser cutting of wood cross sections provides smooth and partially carbonized surfaces with fully open-cell lumina.^80–82^ Unlike mechanical cutting methods, laser cutting applies no mechanical force, enabling intricate designs, narrower kerf widths, and smoother surfaces.^43^ Ding et al. demonstrated that thin wood cross-section membranes fabricated with a commercial CO_2_ laser had fully open, intact cell lumina on both sides.^83^ The developed process has been applied to various wood species, including spruce,^83^ poplar,^84^ balsa, and European yew^85^ (Figure 4f–m). The state-of-the-art thickness of the cut wood is in the range of a few centimeters.

Adding a chemical treatment, the open and aligned lumina channels of wood can be utilized as a scaffold to enable novel functions in wood. For instance, Ding et al. utilized the laser-cut thin cross sections of wood for a chemical treatment to introduce a distinct wettability gradient across the thickness of the wood membrane (e.g., Janus wood membrane [Figure 4e]).^83^ The natural cell lumina channels were utilized as a path for liquid transport. Making use of the driving forces from the wettability difference across the membrane, the wood-based Janus membrane demonstrated directional and spontaneous transport of water.

### Laser incising

Laser drilling, or laser incising, enhances wood permeability, improving penetration, for instance, of wood preservatives. Effective impregnation requires longitudinal and transverse flow of chemical agents.^87^ Traditional approaches to improving permeability include steam, microwave, ultrasonic, biological, mechanical, and laser incision treatments.^88–95^ Among these, laser incision is characterized by precision and flexibility.^25,96–99^

**Figure 5:**
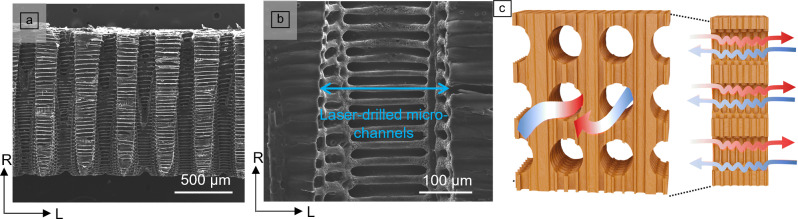
(a, b) Scanning electron microscopy images of laser-drilled wood scaffold. (c) Illustration of a laser-drilled wood panel with enhanced mass exchange (published under license CC BY 3.0).^100^

During laser incising, well-defined holes can be created perpendicular to the wood fiber direction, resulting in three-dimensional interconnected wood scaffolds composed of laser-drilled channels and natural cell lumina.^84,100–103^ Incision efficiency depends on wood species, density, moisture content, laser parameters, and the incision pattern.^45,104,105^ Nath et al. reported successful laser incision in Southern yellow pine and Redwood using CO_2_ and Nd:YAG lasers, achieving controlled incision depths (6–15 mm) and hole diameters (0.80–1.20 mm).^106^ Ding et al. demonstrated controlled CO_2_ laser drilling in the radial direction of wood sections, producing channels ranging from 50 µm to 2 mm, facilitating enhanced mass transport perpendicular to the fiber direction (**Figure** 5a).^100,102,106^ The surrounding cell wall of the laser-drilled channel yielded a char layer (Figure 5b).

Laser drilling has thus opened possibilities to produce highly permeable wood scaffolds.^107^ This interconnected structure significantly promotes mass exchange, improving chemical modification efficiency by allowing deeper impregnation.^25^ Nath et al. demonstrated controlled incision depths and diameters using CO_2_ lasers, greatly enhancing chemical uptake in wood.^45,104^ The same group used laser incising to increase antifungal agent uptake and to facilitate resin penetration in wood species like *Douglas* fir and spruce, significantly enhancing durability without substantial structural weakening.^106^

The interconnected porous structure can increase the moisture/air exchange speed with the atmosphere (Figure 5c).^100,108^ Followed by a chemical treatment with hygroscopic salt, CaCl_2_, the resulting wood displayed superior water adsorption capacity and higher moisture exchange rate. This functionalized wood may support the autonomous regulation of indoor climate, without additional energy input.

This scaffold can also be utilized to introduce geometrical anchoring and interlocking. Ding et al. reported a smart gating wood membrane with thermo-responsive pore actuation by *in situ* polymerized thermo-responsive polymers (PNIPAM) into a laser-drilled poplar wood scaffold.^84^ The laser-drilled wood scaffold acts as a geometric boundary, ensuring the mechanical stability of the composite.

### Surface modification and functionalization

To enhance the environmental sustainability and broaden the practical applications of wood materials, robust and functionally tailored surface properties are crucial. One persistent challenge is the typically weak interface between lignocellulosic substrates and applied coatings.^109–111^ Laser-based surface modification techniques offer precise, localized surface treatments capable of overcoming limitations associated with traditional thermal or chemical methods. Lasering can induce controlled photothermal and photochemical transitions at substrate surfaces, facilitating specific thermochemical reactions without compromising the bulk material integrity.

#### Surface texturing, cleaning, and coloring

Surface texturing, achievable through sub-ablation energy laser scanning, slightly chars and pits the wood surface, thus increasing surface roughness and modifying surface chemistry beneficially for subsequent coatings or composite applications.^112,113^ Nd:YAG lasers (532 nm or 1064 nm) have been successfully employed to remove dirt, varnish, and soot from historical wooden artifacts, revealing original paint layers without damaging underlying delicate wood structures by selectively removing contaminant layers.^114–117^ Another application is controlled wood surface color modification using defocused CO_2_ lasers. Adjusting laser fluence induces thermochemical reactions of wood's lignin and polysaccharides, altering color without additional pigments.^26,118,119^ This charring yields a spectrum of shades from light brown to near-black, enabling applications in decorative branding and improved UV resistance.

#### Wettability tuning

By creating a microtextured and carbonized surface, laser treatment represents a novel wettability tuning method.^120–124^ For instance, femtosecond lasers have been reported to fabricate bioinspired nano-/microstructures mimicking lotus-leaf surfaces on wood and cellulose substrates, significantly altering wetting behaviors toward superhydrophobicity.^114,115,125^ Maciak et al. studied the effects of beam power and feed rate in the process of fresh wood with a CO_2_ laser on the wettability of the cut surface.^126^ Several tree species, such as oak, birch, alder, plum, apple tree, and pine, were investigated. Results showed that CO_2_‑laser cutting can transform wood surfaces from instantly wettable to temporarily water‑repellent by fusing a thin char/crystalline layer.

### Laser-induced carbonization

Laser-induced carbonization (LIC) is a transformative technique that has gained increasing attention for its ability to convert wood^107,127–131^ and other organic substrates^132–137^ into porous, conductive carbonaceous materials using a laser as a localized heat source. In this article, carbonization refers to pyrolysis of the organic precursor into a carbon-rich solid (char), whereas graphitization denotes the development of higher *sp*^2^ ordering within the carbonized product. Unlike conventional thermal carbonization and graphitization methods, which require extremely high temperatures (typically between 1200 and 3000°C) and controlled atmospheres, LIC offers a more accessible and sustainable alternative by eliminating the need for such conditions.^127,128^

The terminology used to describe laser‑derived carbon materials is not uniform across the literature and often reflects the specific properties emphasized by individual studies, such as electrical conductivity, structural features (e.g., porosity), or the degree of *sp*^2^ ordering. Accordingly, terms such as laser‑induced graphene (LIG) are frequently employed to describe *sp*^2^‑rich, highly graphitic carbon networks, rather than the formation of ideal, single‑layer graphene. In this article, laser‑induced carbonization (LIC) is used as a general umbrella term for laser‑induced pyrolytic conversion of organic precursors, while LIG denotes a subset of LIC‑derived materials that exhibit pronounced graphite‑like structural features leading to high electrical performance.

Compared with other biomass-to-carbon conversion routes used for surface or bulk conversion, each method offers distinct strengths. Flame treatment is a simple and cost-effective approach for rapid, large-area surface carbonization of wood and can produce robust, strongly light-absorbing carbonized layers.^138,139^ In contrast, conventional furnace pyrolysis is better suited for high-volume bulk carbon production and can yield graphene-like carbons from sustainable biopolymer precursors under inert atmospheres, but it typically lacks spatial selectivity.^140–142^ Relative to these approaches, LIC is distinctive in enabling digitally programmed, spatially selective conversion and patterning, and is therefore best viewed as complementary rather than competing.

Thanks to its simplicity, the widespread availability of different types of lasers, and the broad range of applicable precursors, LIC has attracted growing attention across multiple research fields. Consequently, various terms have emerged to describe these laser-driven transformations. In this review, the term LIC refers to all laser-induced processes, regardless of whether the resulting carbon is amorphous or graphitic. Terms such as laser-induced graphene,^127,137,143^ laser-induced graphitization,^144–147^ laser direct writing,^133,148^ among others, are considered part of LIC. Processes employing metal catalysts, with iron being the most widely studied, to achieve higher degrees of graphitization are specifically referred to as iron-catalyzed laser-induced graphitization (IC-LIG).^136,149–152^

**Figure 6:**
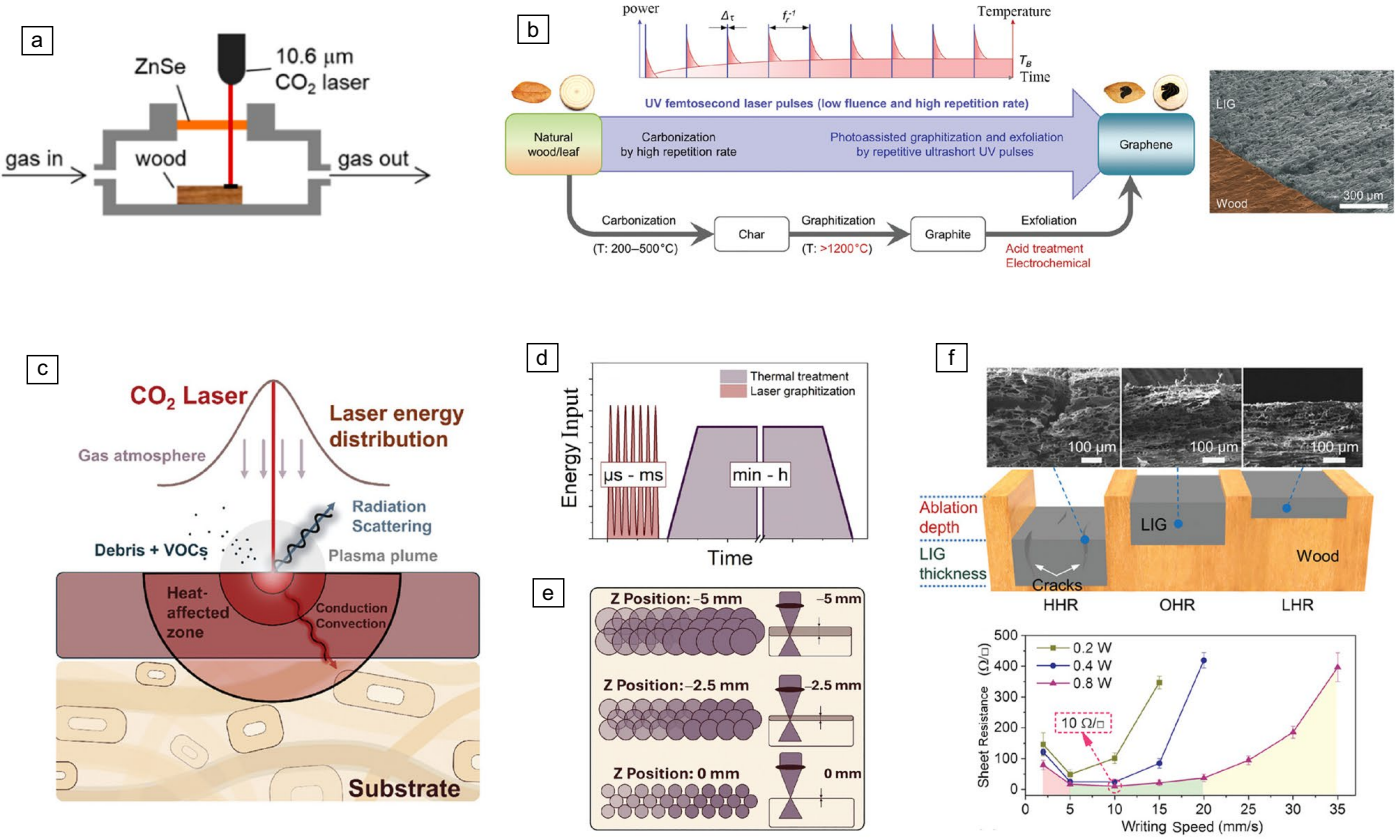
Laser-induced carbonization (LIC) formation on wood and corresponding processing influences. (a) Schematic illustration of wood-derived LIC produced via CO_2_ laser treatment in a controlled atmosphere chamber, enabling laser processing under oxygen-free conditions (photothermal mechanism) (published under license CC BY 4.0).^127^ (b) Schematic of LIC formation from natural wood using a femtosecond laser, based on a photochemical mechanism (© 2019, Wiley).^129^ (c) Summary of laser beam–material interactions, highlighting the Gaussian energy distribution and the resulting heat-affected zone, along with associated effects such as debris formation and volatile organic compound (VOC) release. (d) Comparison between conventional thermal treatments and LIC, emphasizing differences in energy input over time. (e) Focal depth effects on focal area and overlap of the resulting laser-beam diameter (published under license CC BY 4.0).^151^ (f) Impact of writing speed on LIC formation, showing corresponding changes in ablation behavior and crack formation against achieved electrical performance (© 2019, Wiley).^129 LIG, laser-induced graphene; HHR, high heat-accumulation region; OHR, optimized heat-accumulation region; LHR, low heat-accumulation region.^

The concept of laser carbonization dates back to the 1980s;^153^ however, significant progress began in 2014 with the demonstration of CO_2_ laser-induced carbonization of polyimide.^154^ By 2017, the first reports of CO_2_ laser-induced carbonization of wood under an inert atmosphere was published.^127^ Since then, researchers have explored a wide range of precursors and laser sources, ranging from widely commercially available CO_2_ lasers to high-resolution femtosecond laser systems (**Figure** 6a–b), leading to the development of numerous proof-of-concept devices. Organic precursors for LIC include small molecules (e.g., glucose,^155^ citric acid^156^), biopolymers (e.g., cellulose,^132,157^ starch,^136^ lignin,^144^ chitosan,^158^ tannic acid ^159^), and raw biomass such as wood,^107,127–130,160^ bamboo,^161^ cork,^134^ cardboard,^137^ and leaves.^129,162^ Among these, wood stands out as a promising lignocellulosic substrate because of its mechanical integrity and renewability.^128^ However, direct laser conversion of wood into uniform, graphite-like carbon structures with high electrical conductivity remains challenging because of its inherent porosity, anisotropy, and chemical complexity. Research efforts to address these challenges generally follow three main approaches:

1. *LIC on native wood surfaces*, focusing on wood species, laser parameters, and processing conditions
2. *LIC on chemically modified wood*, including chemical impregnation and coatings
3. *Catalytic graphitization during LIC*, using metal catalysts to enhance processing speed and graphitization

#### LIC on native wood surface

In 2017, Ye et al.^127^ reported laser-induced carbonization on the surface of natural, untreated pine wood, which was converted into 3D porous LIC patterns using a 10.6-µm CO_2_ laser. This was achieved by placing small pieces of wood in a controlled atmosphere chamber, allowing control of the Ar/H_2_ flow, while a ZnSe window was mounted on top of the chamber to enable laser irradiation of the wood (Figure 6a). Their studies further showed a direct relationship between applied laser power and the degree of graphitization; higher laser powers resulted in higher conductivities, achieving sheet resistances as low as 10 Ω/sq^−1^.

##### Influence of laser parameters.

The dynamics and efficiency of laser-induced carbonization depend on both the intrinsic properties of the precursor material and the applied laser parameters, including laser power and scanning speed, wavelength, repetition rate, pulse duration, beam shape, focal position, and operational mode (e.g., engraving DPI or cutting) (Figure 6c–e).^151^ Here, it is important to note the different mechanisms associated with the laser source and its wavelength. For long-wavelength lasers such as infrared CO_2_ lasers, the dominant carbonization mechanism is photothermal: absorbed photonic energy is converted into lattice vibrations, generating localized heat that drives structural changes (Figure 6a). In contrast, photochemical reactions are generally associated with short-wavelength lasers, particularly in the ultraviolet range, because their photonic excitation energy matches the resonant range of chemical bond energies, leading to bond breaking (Figure 6b).^163,164^

Dinh Le et al. demonstrated the one-step direct writing of LIC patterns on natural wood using a femtosecond (UV) laser in ambient air.^129^ The high repetition rate of short-wavelength femtosecond laser pulses enabled the direct transformation into highly conductive structures by first converting all wood components into an intermediate amorphous carbon and then successively into LIC. By irradiating the wood with ultrafast pulses, thermal stresses were reduced, minimizing ablation and microcracks (Figure 6f), which resulted in LIC patterns with good electrical conductivity (sheet resistance as low as 10 Ω sq⁻^1^). Subsequent studies showed that NIR femtosecond lasers with ultrashort pulse durations effectively minimize thermal damage and achieve high patterning resolution, with linewidths down to 40 µm.^165,166^

In an attempt to laser-carbonize wood under ambient conditions, Chyan et al. reported a method using multiple laser-engraving steps to form carbonaceous structures directly from natural wood with a CO_2_ laser.^137^ Their strategy involved an initial pre-carbonization of the wood surface, where amorphous carbon was formed on pine and oak by applying a large defocus (>50 mm). Subsequent adjustment of the laser to the appropriate focus (~1 mm) resulted in the conversion to conductive LIC patterns.

Often, the LIC structures contain a range of defects such as grain boundaries and vacancies, and disorder. These defects are commonly reflected by a pronounced Raman D band (I_D_/I_G_) and are intrinsic to the ultrafast laser conversion pathway.^154,167^ While such defect-rich networks can be advantageous in applications that benefit from high surface area and abundant active sites, they can also lead to higher resistivity when continuous *sp*^2^ domains are insufficiently developed. Accordingly, strategies to improve electrical conductivity typically aim to increase *sp*^2^ ordering and domain size while preserving the porous architecture. Laser-process approaches include optimizing energy dose/scan conditions to raise local temperature without excessive oxidation. Post-processing offers an additional lever. Flash Joule heating has been demonstrated to rapidly “heal” LIG’s topological defects in milliseconds while maintaining the overall structure/porosity, reducing I_D_/I_G_ from ~0.84 to ~0.33 and delivering an approximately fivefold conductivity increase.^167^

##### Influence of wood species.

Understanding the influence of wood species, with their structural and chemical variations, provides valuable insights for selecting suitable species and adapting laser parameters to successfully produce LIC.^168^ Lengger et al. investigated LIG formation on wood under ambient conditions without pretreatment.^50^ A total of 46 European and Asian wood species, including softwoods and hardwoods with different structures, were examined in detail. Microscopic analysis revealed,^50^ in agreement with previous studies,^150^ that local variations in carbonization were largely dictated by the wood’s natural structure. Earlywood, with its lower density, experienced high ablation, preventing carbonization and leaving behind only loose carbon residues. In contrast, latewood, being denser, formed compact but irregular carbon structures. Density-dependent thermal conductivity played a key role: denser wood dissipates heat more efficiently, which can stabilize the charring process^169,170^ and lead to less ablation. Results further showed that dense, diffuse-porous wood species (>700 kg m⁻^3^) with high soluble lignin content and low extractive content are favorable for the LIG process.^50^ Besides the density variations, the orientation of the laser relative to the wood grain must be considered, as it impacts heat transport and charring rate, especially given wood’s intrinsic thermal conductivity, which is higher along the grain (longitudinal direction).^170^

Initial attempts to model these effects using molecular dynamics (MD) simulations indicated that higher lignin content can result in favorable carbonaceous structures with fewer defects, suggesting that lignin may play a role in developing *sp*^2^ structures during carbonization.^171^ MD simulations could therefore be a useful tool for understanding LIC and the associated carbonization and graphitization processes. However, one must keep in mind that the complex interactions during lasing cannot be exclusively attributed to the degradation of single molecular units.^172^

Moreover, intrinsic material properties such as heat capacity, thermal conductivity, melting point, and evaporation temperature must be considered. During LIC, the laser functions as a dynamically changing, spatially and temporally localized heat source, inducing anisotropic degradation rather than the uniform, slow heating typical of conventional combustion. This localized heating can cause asymmetric shrinkage and crack formation due to uneven heat transfer, which alters graphitization dynamics and compromises structural integrity. Structural irregularities in wood further affect heat dissipation and the homogeneity of carbon formation, while evaporation and ablation release gases and volatile products that influence surface structure, chemistry, and the local reaction environment, particularly in the presence of reactive gases such as oxygen, ammonia, or hydrogen.^173^

#### LIC on chemically modified wood surfaces

To mitigate issues such as severe crack formation and uncontrolled degradation during laser processing, chemical modification of wood has become an effective strategy. One common approach involves impregnating wood and wood-based materials with fire retardants, such as borate-, ammonium-, or phosphorus-containing compounds.^135^ For example, Chyan et al. demonstrated that boric-acid-treated wood, processed in defocus mode to enable multiple laser passes within a single step, achieved a low sheet resistance of about 8Ω sq^–^^1^.^137^

Similarly, Mulla et al. introduced a coating approach using a lignin-based ink precursor to create conductive LIC structures, achieving electrical properties of 18.6 Ω sq^–^^1^ for spruce and 57.1 Ω sq^–^^1^ for pine.^147^ Their method, termed “lignography,”^174^ employed a printable ink composed of lignin and cellulose to pattern circuits, which were subsequently converted into conductive and homogeneous LIC patterns upon irradiation with a 10.6-µm CO_2_ laser beam.

#### Catalytic graphitization during LIC

To address multiple challenges associated with LIC of lignocellulosic substrates that often require multiple lasing steps, inert atmospheres, and hazardous fire retardants, which still result in high substrate ablation, thermal damage (cracks), and nonhomogeneous electrodes, as well as to improve processing speed and efficiency, Dreimol et al. introduced a new approach called iron-catalyzed laser-induced graphitization (IC-LIG).^149–151^

**Figure 7:**
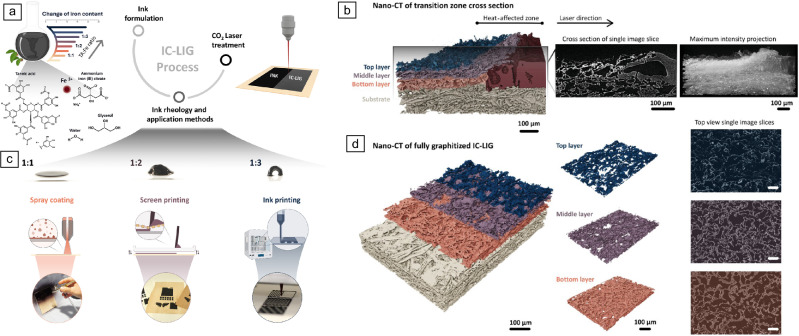
Iron-catalyzed laser-induced graphitization (IC-LIG) approach. (a) Schematic illustration of IC-LIG using CO_2_ laser treatment, showing changes in ink rheology based on varying tannic acid (TA) to Fe^3^⁺ ion ratios (provided via ammonium iron(III) citrate), enabling application techniques such as spray coating, screen printing, and direct ink writing (published under license CC BY 4.0). (b)^149^ Three-dimensional reconstruction of a single nano-computed tomography (CT) image of the transition zone in cross-sectional samples (published under license CC BY 4.0). (c) Including a representative image slice and maximum intensity projection. The 3D reconstruction highlights the transition from native ink to a fully graphitized IC-LIG electrode with distinct layered structure. Top view of a fully graphitized IC-LIG electrode with separated layers, shown alongside the corresponding nano-CT image slice (published under license CC BY 4.0). (d) Scale bar for nano-CT top-view image slices: 100 µm (published under license CC BY 4.0).^151^

Using an iron–tannic acid precursor ink in combination with a conventional CO_2_ laser, they successfully engraved large (≥100 cm^2^), highly conductive (up to 2500 S m^–^^1^) LIG structures on thin (~450 µm) wood veneers and even on paper in a single laser step under ambient conditions (**Figure** 7a).^150^ This method preserved the mechanical integrity of the substrate and significantly enhanced both processing speed and graphitization efficiency, bringing LIC processes closer to industrial scalability.

By adjusting the tannic-acid-to-iron ratio (TA:Fe), the rheological properties of the precursor ink could be tuned, enabling various application techniques such as spray coating, screen printing, and direct ink writing (DIW) (Figure 7b).^149^ Subsequent laser treatment produced functional IC-LIG electrodes across all methods, with even thick DIW-printed layers (260 µm) forming complex, conductive electrode patterns. Laser posttreatment further expanded design flexibility by enabling local tuning of iron phases, for example, converting γ-iron to magnetite.

A multiscale analysis revealed a layered IC-LIG electrode structure consisting of three distinct regions: top, middle, and bottom (Figure 7c–d).^151^ The top and middle layers contained a highly graphitized carbon matrix with an average interlayer spacing of approximately 0.343 nm, while the bottom layer was composed of amorphous carbon. This variation in graphitization was attributed to the unidirectional energy input from the laser. Magnetite was identified as the dominant iron phase across all layers; however, a significant amount of γ-iron was detected in the middle layer, likely due to rapid cooling during laser treatment.

#### Proof-of-concept devices of wood-derived LIC

Wood-derived LIC electrodes have been successfully integrated into a variety of proof-of-concept devices, demonstrating their strong potential for sustainable electronics. Their porous, carbonaceous structure makes them particularly suitable for applications in energy storage, electrocatalysis, sensing, and even smart home technologies.

In the field of energy storage, Ye et al. demonstrated that pine-derived LIC can serve as an effective supercapacitor electrode.^127^ By electrodepositing polyaniline onto the LIC surface, they achieved a specific capacitance of 320 mF cm⁻^2^ at a current density of 10 mA cm⁻^2^, compared to only 1 mF cm⁻^2^ for untreated pine-derived LIC. They also showed that electrodeposition of catalytic materials such as Co-P onto wood-derived LIC can be tuned to facilitate both hydrogen evolution (HER) and oxygen evolution reactions (OERs).

**Figure 8:**
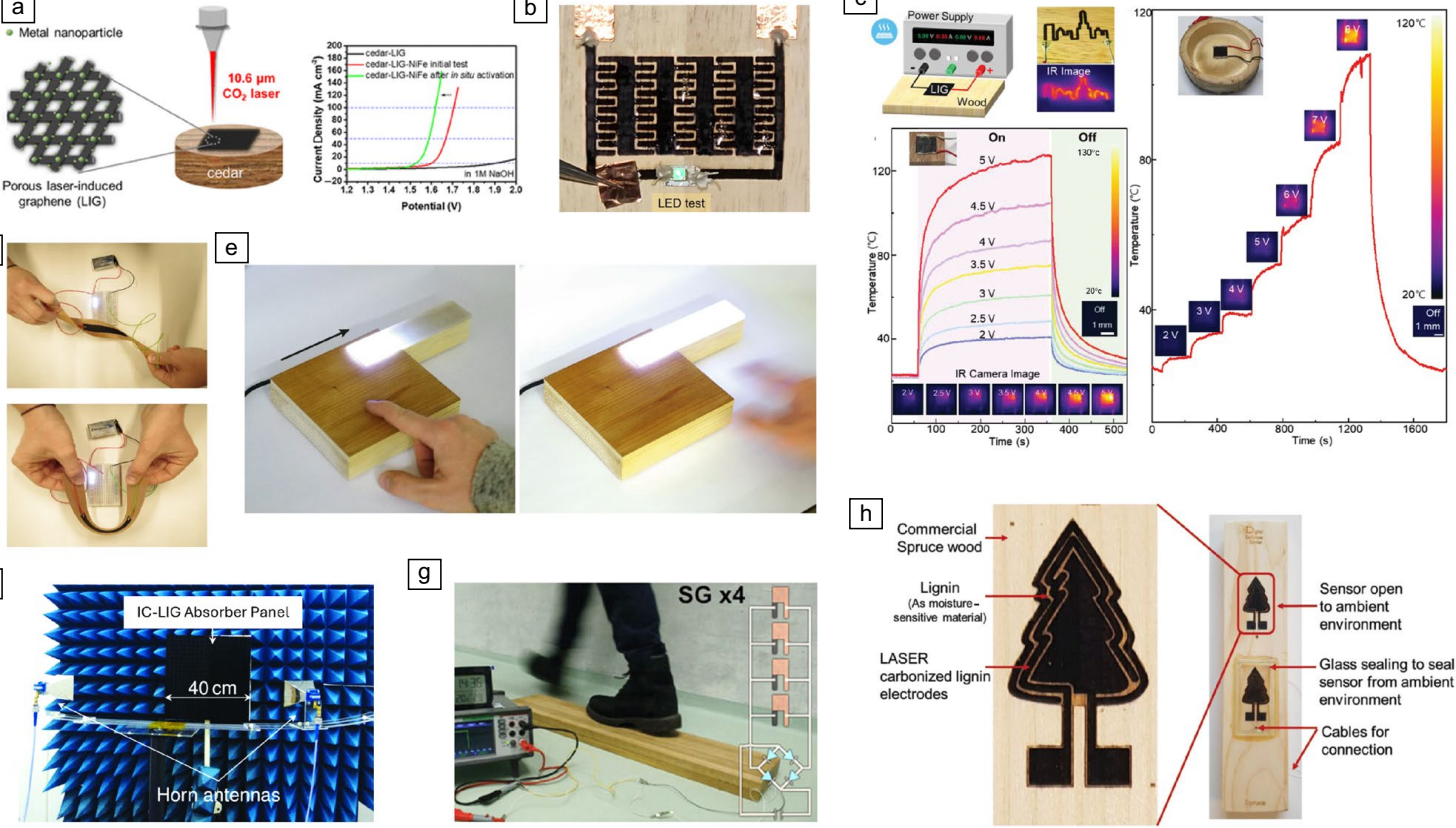
Proof-of-concept devices based on wood-derived laser-induced carbonization (LIC). (a) Schematic illustration of *in situ* formation of metal nanocrystals within LIC via CO_2_ laser treatment of cedar wood soaked with metal nitrate salts, enabling electrocatalytic applications (oxygen evolution reactions) and electromagnetic interference shielding (© 2018, American Chemical Society).^143^ (b) Microsupercapacitor fabricated by embedding manganese monoxide (MnO) into wood-derived LIC using a femtosecond laser (published under license CC BY 4.0).^131^ (c) Schematic of the LIC heater testing setup with corresponding temperature profiles, showing voltage-dependent heating behavior and infrared (IR) images (© 2023, Wiley).^130^ (d) Flexible electrode and (e) touch-sensitive lighting panel based on iron-catalyzed laser-induced graphitization (IC-LIG) electrodes (published under license CC BY 4.0).^128^ (f) Two-layer X-band absorber with resistive patterns engraved on wood using IC-LIG (© 2022, IEEE).^152^ (g) Piezoelectric nanogenerator integrated into a functionalized doorstep, incorporating green piezo-active Rochelle salt in a laser-drilled wood template with IC-LIG electrodes (published under license CC BY 4.0).^107^ (h) Humidity sensor based on spruce wood coated with lignin ink, featuring graphitized interdigitated electrodes separated by non-graphitized lignin acting as a moisture-sensitive layer (published under license CC BY 4.0).^147 LED, light-emitting diode; SG, small gap.^

Han et al.^143^ extended this concept by impregnating wood with metal salts prior to laser processing, thereby creating hybrid LIC structures embedded with inorganic metal nanocrystals (cedar-LIG-M, where M = Cu, Co, Ni, Fe, NiFe) (**Figure **8a). These hybrids were explored for electrocatalytic applications, including OER, as well as for electromagnetic interference (EMI) shielding. Although catalytic graphitization was not explicitly reported, the use of a controlled Ar/H_2_ atmosphere and metal salt infiltration likely enhanced graphitization, which may also explain the low sheet resistance values of 7 Ω sq⁻^1^.

Microsupercapacitors have also been realized by embedding manganese monoxide (MnO) onto wood-derived LIC using the femtosecond laser direct writing (FsLDW) technique (Figure 8b).^131^ This was achieved through a two-step approach: first, the formation of LIC on the wood surface, followed by drop-casting a small amount of MnO precursor. A subsequent laser cycle converted the precursor into MnO, resulting in MnO/LIG hetero nanostructures. These structures exhibited an areal capacitance of 35.54 mF cm⁻^2^ at a scan rate of 10 mV s⁻^1^ and retained approximately 82.31% of their capacitance after 10,000 cycles. The resulting microsupercapacitors were capable of powering small electronic devices such as light-emitting diodes, digital clocks, and electronic paper.

Dreimol et al. introduced robust and flexible electrodes based on iron-catalyzed laser-induced graphitization (IC-LIG), fabricated on thin wood veneers (up to 450 µm) from eight different species.^150^ These electrodes enabled several functional devices, including strain sensors that endured more than 69,000 cycles of tensile loading, touch-sensitive control panels for lighting, and electrodes for electroluminescent devices. The flexibility of the electrodes allowed for twisting and bending without compromising performance (Figure 8d–e). They further demonstrated a large-scale, sustainable X-band absorber (40 × 40 cm), validated through simulations and measurements to operate effectively at incident angles up to 45° off broadside in the 7–12 GHz frequency range (Figure 8f).^152^ Additionally, they demonstrated a nanogenerator device based on a fully recyclable, reusable, and high-performing piezoelectric system resulting in a functionalized doorstep (Figure 8g).^107^ The device incorporates green piezo-active Rochelle salt embedded in a laser-drilled wood template, with IC-LIG based on a shellac incorporated precursor ink used to apply the electrodes. By investigating different crystal pillar configurations, a shearing design with 45°-oriented pillars achieved output voltages of up to 30 V and currents of 4 µA, corresponding to a tenfold increase in power compared to single-crystalline Rochelle salt.

Smart home and smart furniture applications have also been realized using wood-derived LIC via femtosecond-laser direct writing, including resistive heating elements, sensors, and touch interfaces (Figure 8c).^130,175,176^ These examples highlight the potential of LIC for integration into everyday functional materials and devices. The same group presented hydrophobic LIC patterns, showcasing a low sheet resistance of 10.0 Ω⋅sq⁻^1^ and a high contact angle of 148.8°, envisioned for smart wooden roofing solutions.^165^

In the area of sensing, Mulla et al. developed a humidity sensor using wood-derived LIC combined with lignin as the active sensing material (Figure 8h).^147^ The sensor, characterized via impedance spectroscopy (1 Hz–100 kHz) in a controlled climate chamber, operated across a humidity range of 10–90% RH at 25°C. Sensitivity reached 2.6 MΩ/% RH for spruce and 0.74 MΩ/% RH for pine, demonstrating the feasibility of LIC-based sensors for environmental monitoring.

## Perspectives on future development of laser processing of wood materials

In the previous section, we reviewed the state of the art of the mechanism and applications of the laser-enabled wood modifications and functionalization. Although the field has advanced rapidly, laser processing of lignocellulosic substrates is still emerging. In this section, we provide several perspectives on the future development of laser processing of wood materials.

### Unraveling laser–material interaction mechanisms for targeted properties

Understanding how specific laser parameters dictate reaction pathways is essential for property optimization. Previous research showed that different lasers induce distinct regimes. Recent work suggests that vibrationally resonant mid-IR pulses can selectively excite specific bonds (e.g., targeting lignin’s aromatic ether linkages a t ~7 µm to promote depolymerization).^177^ Understanding under which conditions a laser acts as a “photochemical scalpel” versus a heat source is thus a key fundamental question. Addressing this will require systematic studies across wavelengths and pulse lengths.

### Role of chemical compositions

Because wood comprises cellulose, hemicelluloses, lignin, and extractives in heterogeneous distributions, composition strongly influences laser outcomes. Future studies should extend such analyses to chemically modified wood and wood hybrids, where additives may shift absorption spectra and decomposition pathways. Establishing robust chemistry-structure–property relationships will ultimately enable rational precursor design for laser manufacturing of different wood species.

### From traditional lasers to swept lasers

The traditional lasers used in materials processing (e.g., Nd:YAG at 1064 nm, CO_2_ at 10.6 µm, excimer lasers at 248 nm, etc.) all operate at a fixed emission wavelength. Such lasers are highly optimized for power, beam quality, and efficiency at that specific wavelength, and they excel when the chosen wavelength matches the application (e.g., 1064 nm for many metals, 10.6 µm for many organic materials). Therefore, when one laser must address multiple components, compromises arise.

Notably, wood is a heterogeneous natural composite composed of cellulose, hemicellulose, and lignin, each with distinct optical and chemical properties. Moreover, chemicals modification approaches introduce new components to the wood substrates, making the processing even more challenging. Unlike traditional fixed-wavelength lasers, a swept laser can “sweep” across a range of wavelengths during operation. A single swept laser can essentially serve as multiple lasers in one, spanning a range of wavelengths. Swept lasers are emerging as powerful and versatile tools in materials processing, allowing dynamic optimization for different materials and tasks.^178–181^ For complicated biobased materials such as wood, a swept laser could adjust to a wavelength that selectively excites specific bonds or components to allow more precise and targeted processing than fixed-wavelength lasers.

The implementation of swept lasers will require addressing beam-delivery efficiency, component cost/robustness, and throughput tradeoffs versus industrial CO_2_ systems; we therefore present swept-source processing as a promising research direction rather than a near-term production replacement.

### Toward scalable laser manufacturing of wood

Although many laser-enabled wood modifications have been demonstrated at laboratory scale, scalability is ultimately determined by (1) throughput and (2) process robustness to intrinsic variability in wood. On the throughput side, recent LIC/LIG-related work already demonstrated routes toward higher-speed and larger-area processing using industrially relevant CO_2_ laser platforms (e.g., maximum scan rates on the order of m s⁻^1^), and large-area, homogeneous graphitized electrodes have been produced (e.g., ~100 cm^2^).^182^ Importantly, process robustness can be improved upstream by surface homogenization: applying a uniform, laser-active precursor layer can compensate substrate irregularities and reduce sensitivity to anatomical heterogeneity.^151^ Scalable deposition routes such as spray coating have shown particularly good uniformity and reproducibility for large-area coverage, which is directly relevant for roll-to-roll or panel-level manufacturing concepts.

Building on these advances, emerging *in situ* sensing and closed-loop control, combined with process databases and AI models, provide a pathway toward “self-correcting” manufacturing that compensates for wood heterogeneity in real time.^183^ In addition to regulating conventional parameters (power, scan speed, focus), advanced controllers could dynamically tune the emission wavelength $\mathrm{\uplambda }(\mathrm{t})$ and pulse conditions, enabling adaptive spectral programs tailored to heterogeneous substrates while respecting safety and throughput constraints. Lull et al. showed that high-speed imaging coupled with infrared spectroscopy can capture millisecond-scale pyrolysis events in individual wood fibers.^184^ Embedding such solutions could enable real-time parameter adaptation for variable feedstocks and supporting scalable implementation.

## Conclusion

This article highlights laser processing as an enabling approach for the functionalization and engineering of wood materials, offering clear advantages over conventional methods in terms of precision, versatility, and digitally defined, localized modification. By leveraging distinct laser–material interaction regimes, laser processing enables wood modification across multiple length scales, from cutting and drilling to surface functionalization and localized carbonization. Emerging opportunities, most notably laser-induced carbon materials, illustrate the potential of lasers to transform wood from a structural resource into a functional platform for electronics, sensing, and energy-related devices.

The most immediate application potential lies in high-precision machining of wood and composites, surface engineering to improve adhesion and wettability control, and integration of conductive carbon patterns for “smart wood” components. However, broader commercialization will depend on addressing two persistent challenges: (1) controlling the heat-affected zone and related safety/emissions issues, and (2) managing wood’s intrinsic heterogeneity to achieve reproducible outcomes at industrial relevant scale. Future developments should therefore prioritize systematic elucidation of laser–wood interaction and composition–structure–property relationships, together with advances in laser setups and *in situ* monitoring/closed-loop control that can adapt processing conditions to variable feedstocks. Overall, laser processing represents a powerful toolbox for scalable, tailored wood functionalization aligned with evolving engineering and sustainability demands.
